# Epidemiology of hepatitis C: related hepatocellular carcinoma in Cameroon

**DOI:** 10.11604/pamj.2014.19.379.4143

**Published:** 2014-12-15

**Authors:** Firmin Ankouane Andoulo, Dominique Noah Noah, Roger Djapa, Mathurin Kowo, Paul Talla, Edith Hell Medjo, Isidore Kamsi Djomkam, Bernadette Ngo Nonga, Oudou Njoya, Elie Claude Ndjitoyap Ndam

**Affiliations:** 1 Yaounde University Hospital Center, Department of internal medicine and specialties, Faculty of medicine and biomedical sciences, University of Yaounde I, Yaoundé, Cameroon; 2 Yaounde Central Hospital, Department of internal medicine and specialties, Faculty of medicine and pharmaceuticals sciences, University of Douala, Douala, Cameroon; 3 Yaounde General Hospital, Department of internal medicine and specialties, Faculty of medicine and biomedical sciences, University of Yaounde I, Yaoundé, Cameroon; 4 Yaounde Central Hospital, Department of Radiology, Faculty of medicine and biomedical sciences, University of Yaounde I, Yaoundé, Cameroon; 5 Yaounde University Hospital Center, Department of surgery and specialties, Faculty of medicine and biomedical sciences, University of Yaounde I, Yaoundé, Cameroon

**Keywords:** Hepatocellular carcinoma, hepatitis C, developing countries, epidemiology, cirrhosis, alcohol, parenteral exposition, Cameroon

## Abstract

**Introduction:**

Hepatocellular carcinoma (HCC) is a global public health problem. Hepatitis C virus (HCV) infection accounts for close to 24% of HCC in developing countries especially when associated with cirrhosis. There exists no vaccine against HCV to prevent the occurrence of HCV-related HCC. A sound knowledge of the epidemiology and prevention of the initial infection is vital. The aim of our study was to determine the epidemiologic profile of HCV-related HCC in Cameroon to improve its’ management.

**Methods:**

It was a prospective study of histologically proven HCV-related HCC seen in two University Centers in Yaounde, Cameroon from March 2012 to January 2013. Demographic data (age, gender), alcohol abuse (>80g/day), presence of cirrhosis, tobacco abuse and parenteral exposition were analyzed.

**Results:**

Twenty-six patients with histologically proven HCV–related HCC were included (18 men (69.2%) and 8 women (30.8%); mean age +/- SD, 61.46+/-10.18 years). A total of 22 (84.6%) patients had a parenteral exposition, 02 (7.7%) patients were alcoholics and 06 (23.1%) patients were smokers. The proportion of patients with cirrhosis was 69.2% against 30.8% cirrhosis-free. Patients with cirrhosis were relatively younger than those cirrhosis-free (mean age +/- SD, 59.05+/-10.05 years vs 66.87+/- 8.72 years, p=0.06). HCV-related HCC was more prevalent in 60 years and above patients (53.8%, 95%CI: 33.4-73.4). The relative risk of HCC among alcoholics patients was high (RR: 1.5, 95%CI: 1.13-1.99, p<0.05).

**Conclusion:**

In Cameroon, HCV-related HCC is more prevalent among age older than 60 years, a finding which is relatively less to that found in western countries, male gender is twice more at risk than female gender and cirrhosis frequency is less compared to that observed elsewhere. HCV and alcohol play a synergistic role in the occurrence of HCC in our environment.

## Introduction

Primary liver cancer, particularly hepatocellular carcinoma (HCC) is a major public health problem at a global scale [1]. It ranks among the three major causes of cancer-related deaths in the world [2, 3]. The incidence of HCC varies with respect to demographic characteristics (gender, age, and race/ethnicity), geographic region and etiologic factors [3–5]. As a matter of facts, there exists a risk stratification according to geographic region (high risk, intermediate risk and low risk) and diverse etiologic factors, most especially hepatitis virus B and C infection appear to play a predominant role in its’ occurrence worldwide [1–3, 5, 6]. Other non-infectious agents (such as diabetes, alcohol, smoking, aflatoxin and obesity) are known to play a role in the occurrence of HCC [3, 5]. Studies in developed countries came out with the conclusion that HCC mostly affects aged patients, preferentially men and cirrhotic patients [1, 7]. The role played by hepatitis C virus (HCV) in the pathogenesis of HCC and the epidemiologic characteristics of patients are poorly understood in Cameroon. It has been estimated that close to 24% of cases of HCC are related to HCV in developing countries against 50% to 70% in southern Europe and Japan (regions considered at intermediate risk) [7, 8]. A sound knowledge of the epidemiologic characteristics of patients with HCC and the prevention of the initial infection are vital in preventing the occurrence of HCC [4, 9, 10]. In Cameroon, as is the case with most developing countries, preventive methods against blood borne HCV infection was not effective during the 1980s. The aim of our study was to determine the epidemiologic profile of HCV-related HCC in Cameroon, among blacks, in order to improve its’ management.

## Methods

A total of 30 patients with histologically proven HCC and infected with HCV were enrolled at the University Hospital Center and Yaounde General Hospital, Cameroon between March 2012 and January 2013. Demographic data (age, gender), alcohol abuse (>80g/day), smoking, presence or absence of cirrhosis and parenteral exposition were entered in a data entry form by a resident in internal medicine. HCV antibodies were tested using a 3^rd^ generation ELISA test, using the commercial kit Fortress^®^ diagnostics, Northern Ireland, United Kingdom. Radiologic (ultrasonography and computed tomodensitometry) and histopathologic criteria were used to establish the diagnosis of HCC. Cirrhosis was diagnosed based on radiologic criteria performed by a single radiologist and confirmed by histopathologic criteria. Exclusion criteria were cases of co-infection (either with HBV or HIV), doubtful diagnosis of HCC and cirrhosis. Data was analyzed using the French version of Epi info 6.04 and Excel 2007. For quantitative variables, means and standard deviations were calculated. For qualitative variables, proportions were calculated with their confidence intervals (CI). To examine the relationship between two discrete variables, we used Pearson's χ^2^ test. Yates correction and Fischer's exact test were used for small sample sizes, with a p value set at 0.05. Kruskall Wallis test was used to compare the average ages of cirrhotic and non cirrhotic patients.

## Results

A total of 30 patients with histologically proven HCC were identified of which four were excluded given their co-infection with hepatitis B virus. Twenty-six patients met our inclusion criteria. The mean age was 61.46±10.18 years (extremes 38-80 years) and 69.2% (18/26) were men (sex ratio 2.3:1). Cirrhosis was found in 69.2% (18/26) of patients against 30.8% (8/26) who were cirrhosis-free. Also, 23.1% (6/26) of patients were smokers while 7.7% (2/26) of patients were alcoholics. The prevalence of HCC with respect to age was 0% among those aged < 0years; 3.8% (95%CI: 0.1-19.6) in 30-39 years; 3.8% (95%CI: 0.1-19.6) in 40-49 years; 38.5% (95% CI: 20.2-59.4) in 50-59 years and 53.8% (95%CI: 33.4-73.4) in age=60 years. Parenteral exposition other than injection drug usage was encountered in 84.6% (22/26) of patients (Table 1).

**Table 1:** Epidemiologic characteristics of patients with HCV-related hepatocellular carcinoma in Cameroon

variables	Number of cases	(%)	95%CI
**Age (years)**			
<30	0	-	-
30-39	1	3.8	0.1-19.6
40-49	1	3.8	0.1-19.6
50-59	10	38.5	20.2-59.4
≥60	14	53.8	33.4-73.4
**Gender**			
Male	18	69.2	48.2-85.7
Female	8	30.8	14.3-51.8
**Cirrhosis**			
Yes	18	69.2	48.2-85.7
No	8	30.8	14.3-51.8
Parenteralexposition*			
Yes	22	84.6	70.7-98.4
No	4	15.4	1.6-29.3

* Parenteral exposition other than injection drug usage (tatooing, scarifications, piercing, blood and its derivatives, dental care, surgery). CI confidence interval. % percentage

Table 2 presents the epidemiologic characteristics of HCV-related HCC both among patients with and without cirrhosis. Patients with cirrhosis were relatively younger than those without cirrhosis (mean age +/- SD, 59.05+/-10.05 versus 66.87+/-8.72 years, p=0.066). The presence of cirrhosis diminished gradually with increasing age (100% at 30-39 years to 57.0% at = 60 years), cirrhosis-free patients were more frequent as from 50 years old. Cirrhosis was more frequent among women (87.5%), relative risk (RR) (RR 1.43 CI 95%:0.91-2.25), among alcoholics (100%) (RR 1.5 CI 95%: 1.13-1.99) and non smokers (70.0%) (RR 0.95 IC 95%:0.51-1.80 for the smoker).

**Table 2:** Characteristics of HCV-related hepatocellular carcinoma among patients with cirrhosis and cirrhosis-free patients

Variables	Effective	Patient with cirrhosis		Cirrhosis-free patients		p-value
		Number of cases	Percentage (%)	Number of cases	Percentage (%)	
**Mean age (years)**		59.05±10.05		66.87±8.72		0.066
**Age (years)**						
<30	0	0	-	0	-	
30-39	1	1	100	0	-	
40-49	1	1	100	0	-	
50-59	10	8	80.0	2	20.0	
≥60	14	8	57.0	6	42.9	0.49
**Gender**						
Male	18	11	61.1	7	38.9	
Female	8	7	87.5	1	12.5	0.36
**Alcohol**						
Yes	2	2	100	0	-	
No	24	16	66.7	8	33.3	1.00
**Tobacco**						
Yes	6	4	66.7	2	33.3	
No	20	14	70.0	6	30.0	1.00

## Discussion

Hepatocellular carcinoma (HCC) ranks among the most frequent causes of cancer-related deaths worldwide [1, 2, 5, 6]. Hepatitis C viral infection is a known independent risk factor for the development of HCC [11], a factor clearly established especially in countries of intermediate prevalence of HCC such as southern Europe and Japan [12] where the epidemiology of HCV-related HCC has been sufficiently described. Lack of data in our setting prompted us to carry out this study in Cameroon. In this study, the characteristics of 26 patients with histologically proven HCV-related HCC were analyzed. Our results show that patients who rank first include patients beyond age 50 (mean age: 61.5 years), who present with cirrhosis and have a parenteral exposition. Equally, male gender is twice more concerned than female gender. Last, but not the least, there exist a synergistic role between alcohol abuse and HCV infection in the pathogenesis of HCC in our country.

These factors for long have been analyzed by several epidemiological studies. Results from these studies are similar to ours bet for some differences. Firstly, the prevalence of cirrhosis was relatively lower among our patients (69.2% vs 90%). HCC on a cirrhosis-free liver in our setting is more associated with hepatitis B virus whose oncogenic properties are clearly established [13, 14]. Patients with cirrhosis were paradoxically found to be relatively younger and had a lower risk of developing HCC than aged patients with cirrhosis. Both clinical and experimental studies have clearly established the positive correlation between HCV infection and the incidence of HCC in patients with cirrhosis in southern Europe and Japan [6, 14, 15]. More so, cirrhosis, whatever it's cause, is a major risk factors for the development of HCC [7, 13]. Among young patients in our study, this finding was not verified. This can be accounted for by the short life span of these young patients with cirrhosis as a result of their inadequate management [7]. As a result, they do not have sufficient time to develop HCC than aged patients, despite the fact that they are more affected by cirrhosis. Lastly, the mean age of patients with HCV-related HCC is lower than that found in western countries [5].

The synergistic role of alcohol abuse and HCV infection in the development of HCC has been documented in many studies [15–17], amongst which that of Kumar et al [18]. According to authors, this synergy is due to the presence of viral RNA and not the presence of anti-HCV. Our study showed that all alcoholic patients with HCC had cirrhosis and were more at risk than did non alcoholics. Given its cost, viral RNA was not detected in our patients. There is a likelihood of detecting viral RNA among these alcoholic patients. Parenteral exposition was found in our study, with the difference being that it was more nosocomial (surgery, dental care, blood transfusions, and abortions) and traditional (scarifications, tattooing and piercing). Injection drogue usage was not found. This parenteral exposition in our setting can be accounted for by the absence of measures preventing contamination through blood and its derivatives during the 1980s on one hand, and by the scarcity of prevention of viral hepatitis transmission programs in developing countries due to financial constraints on the other hand [3].

## Conclusion

In Cameroon, age older than 60 years, male gender, past history of a parenteral exposition and cirrhosis are characteristics of most patients with HCV-related HCC. Alcohol has a synergistic role with HCV in the pathogenesis of HCC in our setting. In the absence of a vaccine against HCV, preventing HCV-related HCC requires preventing the initial viral infection and clearing the infection using antiviral therapy.
